# P-369. Safety and Efficacy of Doravirine/Islatravir (DOR/ISL) 100/0.25 mg Once Daily (QD) after ISL Dose Reduction from 0.75 mg: Week 48 Results from an Open-Label Phase 3 Study

**DOI:** 10.1093/ofid/ofaf695.587

**Published:** 2026-01-11

**Authors:** Rosie Mngqibisa, Sheetal Kassim, Cheryl McDonald, Mark Bloch, Eugenie Colin-Benoit, Christopher Bettacchi, Margaret Johnson, Hiroyuki Gatanaga, Euna Kim, Uche Nwoke, Michelle C Fox, Luisa M Stamm, Mengchun Li, Wayne Greaves

**Affiliations:** Enhancing Care Foundation, Durban, KwaZulu-Natal, South Africa; Desmond Tutu Health Foundation, Cape Town, Western Cape, South Africa; Tarrant County Infectious Disease Associate, Fort Worth, TX; Holdsworth House, Syndey, New South Wales, Australia; Department of Infectious Diseases, Inselspital, Bern University Hospital, University of Bern, Bern, Bern, Switzerland; North Texas Infectious Disease Consultants, Dallas, Texas; Royal Free London NHS Foundation Trust, London, England, United Kingdom; National Center for Global Health and Medicine, Shinjuku-ku, Tokyo, Japan; Merck & Co., Inc., Rahway, New Jersey; Merck & Co., Inc., Rahway, New Jersey; Merck & Co., Inc., Rahway, New Jersey; Merck & Co., Inc., Rahway, New Jersey; Merck & Co., Inc., Rahway, New Jersey; Merck Research Labs, Edison, NJ

## Abstract

**Background:**

Phase 2 and Phase 3 studies of DOR/ISL 100/0.75 mg QD demonstrated good antiretroviral activity in adults with HIV-1. However, declines in total lymphocyte and/or CD4+ T-cell counts were observed. Modeling studies predicted that ISL 0.25 mg would achieve efficacy comparable to ISL 0.75 mg with no meaningful declines in total lymphocyte or CD4+ T-cell counts. We studied the safety and efficacy of DOR/ISL 100/0.25 mg in participants who had received DOR/ISL 100/0.75 mg previously.

**Table 1.**
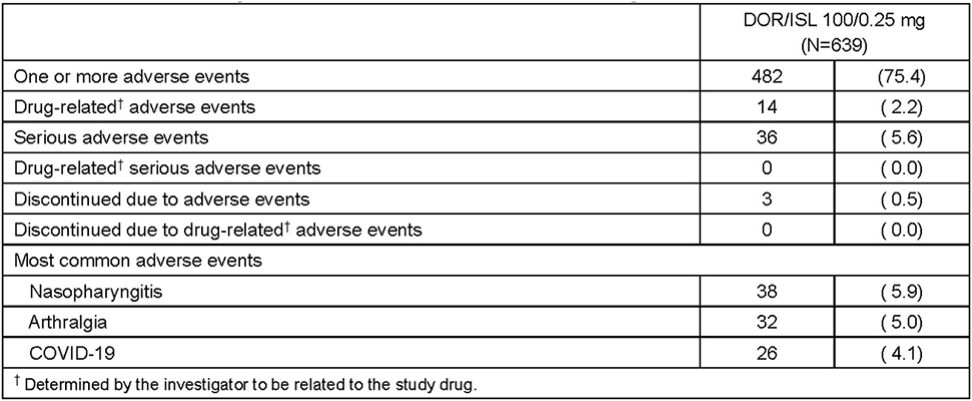
Summary of Adverse Events through Week 48

**Table 2.**
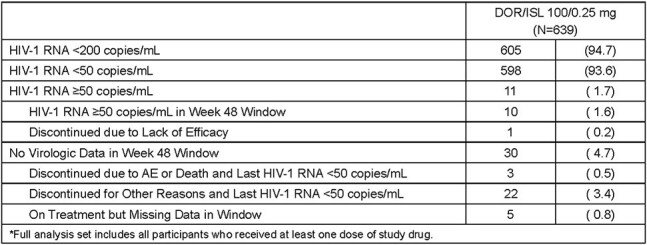
Virologic Outcomes at Week 48 (FDA Snapshot Approach; Full Analysis Set*)

**Methods:**

In this phase 3, open-label, single-arm study (NCT05766501), adults with HIV-1 RNA < 200 copies/mL who had tolerated DOR/ISL 100/0.75 mg QD in a previous study (MK8591A-018, 020, or 033) switched to open-label DOR/ISL 100/0.25 mg QD for maintenance therapy. The primary objective was to evaluate the safety and tolerability of DOR/ISL 100/0.25 mg QD through week 96. Secondary objectives included assessment of the antiretroviral activity of DOR/ISL (FDA Snapshot Approach) and changes in total lymphocyte and CD4+ T-cell counts. An interim analysis conducted at week 48 is reported here.

**Figure 1.**
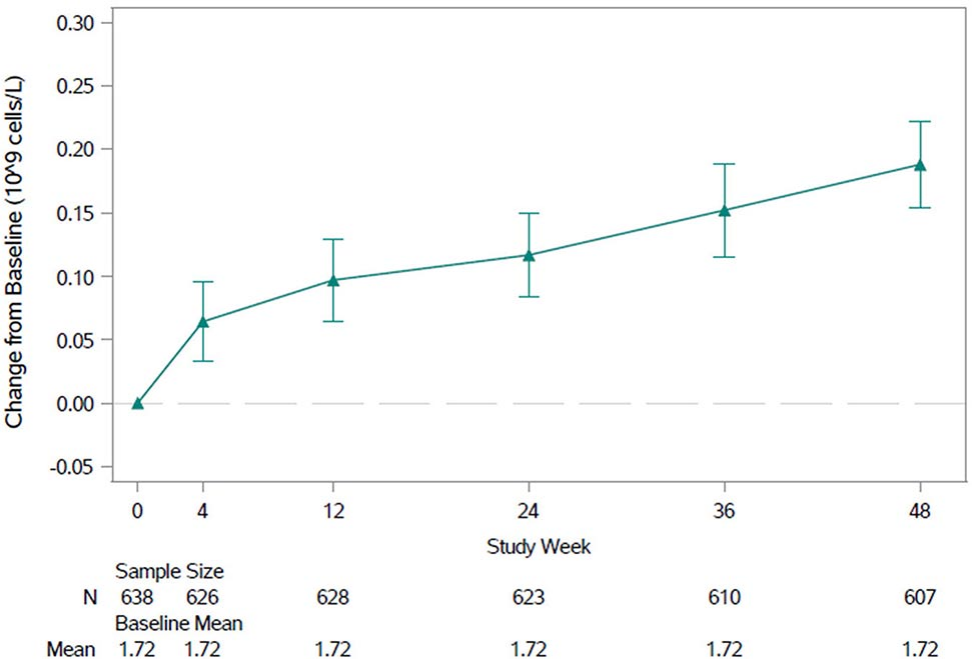
Total Lymphocyte Count, Mean Change from Baseline (and 95% CI)

**Figure 2.**
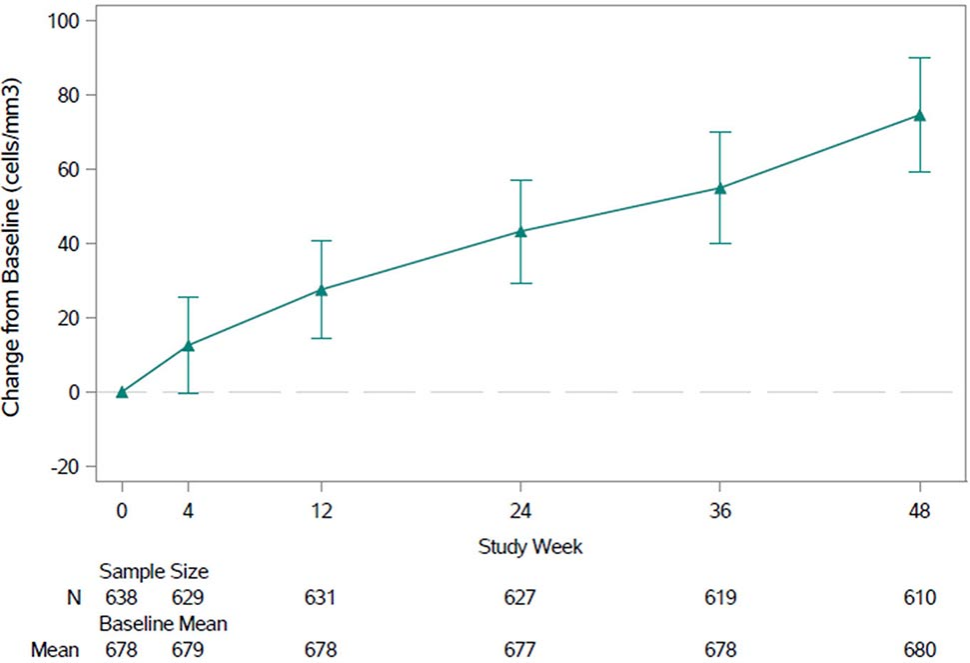
CD4+ T-cell Count, Mean Change from Baseline (and 95% CI)

**Results:**

639 participants entered the study: mean age 44.7 (range 20-79) years, 69.3% male, 55.7% white, and 31.5% black/African American. Mean time on DOR/ISL before enrollment was 31.7 (range 17.6-61.9) months. By week 48, adverse events (AE) were reported by 75.4% of participants and were considered drug-related in only 2.2% (Table 1). Serious AEs were reported in 5.6% of participants; none were drug-related. Three participants (0.5%) discontinued treatment due to an AE; none were drug-related. At week 48, HIV-1 RNA was < 50 copies/mL in 598 participants (93.6%) and ≥50 copies/mL in 11 (1.7%) (Table 2). Two participants (0.3%) developed clinically significant viremia (confirmed HIV-1 RNA ≥200 copies/mL); resistance data were not available due to assay failure. Total lymphocyte and CD4+ T-cell counts showed mean increases from baseline of 0.19x10^9^ cells/L (95% CI: 0.15, 0.22) and 74.5 cells/mm^3^ (95% CI: 59.2, 89.8), respectively, at week 48 (Figures 1 and 2).

**Conclusion:**

DOR/ISL 100/0.25 mg QD was generally well tolerated and maintained virologic suppression in a high proportion of participants who had previously received DOR/ISL 100/0.75 mg. Baseline total lymphocyte and CD4+ T-cell counts increased after ISL dose reduction.

**Disclosures:**

Cheryl McDonald, MD, Gilead: Advisor/Consultant|Gilead: Grant/Research Support|Merck: Grant/Research Support|Viiv: Grant/Research Support Mark Bloch, MBBS, MMed, Gilead Sciences: Lectures|GSK: Board Member|GSK: Lectures|ViiV Healthcare: Advisor/Consultant|ViiV Healthcare: Lectures, Support to attend scientific meetings Christopher Bettacchi, MD, Merck: Honoraria|Viiv: Honoraria Margaret Johnson, MD, Merck & Co., Inc.: Grant/Research Support Euna Kim, BA, Merck & Co., Inc.: Stocks/Bonds (Public Company) Uche Nwoke, MS, Merck & Co., Inc.: Stocks/Bonds (Public Company) Michelle C. Fox, MD, Merck & Co., Inc.: Employment|Merck & Co., Inc.: Stocks/Bonds (Public Company) Luisa M. Stamm, MD, PhD, Merck & Co., Inc.: Employment|Merck & Co., Inc.: Stocks/Bonds (Public Company) Mengchun Li, MD, Merck & Co., Inc.: Stocks/Bonds (Public Company) Wayne Greaves, MD, Merck & Co., Inc.: Stocks/Bonds (Public Company)

